# “FGM” vs. female “cosmetic” surgeries: why do they continue to be treated separately?

**DOI:** 10.1038/s41443-021-00514-8

**Published:** 2021-12-15

**Authors:** Arianne Shahvisi

**Affiliations:** Brighton and Sussex Medical School, Brighton, BN1 9PX UK

**Keywords:** Surgery, Health care

## Abstract

In this article, I argue that the moral and legal distinction between “female genital cutting” and “female genital cosmetic surgeries” cannot be maintained without recourse to racist distinctions between the consent capacities of white women and women of colour. The physical procedures involved in these surgeries have significant overlap, as do their motivations, yet they are treated differently in everyday discourse and the law. This paper lays bare this double standard and presents and interrogates some of the reasons commonly given to justify their separate treatment. It concludes with the recommendation that the distinction be dropped in favour of more consistent consent-based stance, which avoids the racism and ethnocentrism that underwrites the present regime. According to this position, the only defensible moral and legal distinction is between those who can consent to these procedures, and those who cannot.

## Introduction

The set of practices commonly grouped under the heading “female genital mutilation” (FGM) are widely reviled in Western contexts, where they are commonly presented as a form of gender-based violence and child abuse (see e.g. [1]). “FGM” was recently described by a UK judge as “a barbaric and sickening crime” [2], and the World Health Organisation describes these practices as “a violation of the human rights of girls and women and as an extreme form of gender discrimination” [3]. By contrast, practices known as “female genital cosmetic surgery,” which are becoming more common, are generally deemed to be a matter of individual choice. The difference in framing is codified within most Western legal systems. In the UK, “FGM” is unlawful even for adult women, while nonmedical “FGCS” is lawful, even for minors, on the grounds that it responds to a mental health need [4]. This differential treatment is problematic because the procedures classified as “FGM” and FGCS have significant overlap both in their physical details and their social functions. Here, I present, problematise, and explain this double standard, and suggest how we might move beyond it.

Before I begin, several points on scope and terminology should be noted. First, I refer to “female genital cutting” instead of “female genital mutilation,” because: (a) value-laden terminology tends to foreshadow discussions of genital cutting, and while some people consider their genitals to have been mutilated, others view cutting more neutrally, or even as an enhancement; (b) it is more consistent with the neutral terms used for other forms of genital modification, i.e. “male circumcision,” “intersex surgery,” and “female genital cosmetic surgery.” Second, I use the term “Western” to refer to Europe, North America, and Australasia, and “non-Western” to refer to all other contexts. This is a troubling dichotomy (see e.g. [5]), but one of the key points of this paper is that the morally suspect distinction between FGC and FGCS rests on a morally suspect division between “the West” and “the rest,” and so I adopt the language that is needed to make this argument. Third, I take the UK context as the focus of my analysis, though most Western jurisdictions exhibit similar contradictions. Finally, I am concerned only with genital cutting for *nonmedical* reasons. I therefore exclude cases in which the cutting is made in accordance with evidence-based medical practice in order to attend to an existing or impending threat to wellbeing posed by diseased or dysfunctional tissue.

## Similarities between FGC and FGCS

Neither FGC nor FGCS refers to a single practice; both are umbrella terms for a wide range of modifications which range in severity from piercing or pricking the clitoris or labia to removing most of the external clitoral and labial tissue and suturing the vaginal opening. It is therefore important, when encountering these terms, to establish the precise details of the practice under discussion. Attention must also be paid to differences in the motivations of those who request the cutting, its prevalence, and the consent status of the person whose genitals are cut. These details ought to be relevant to any moral or legal response to these practices.

**Fig. 1:**
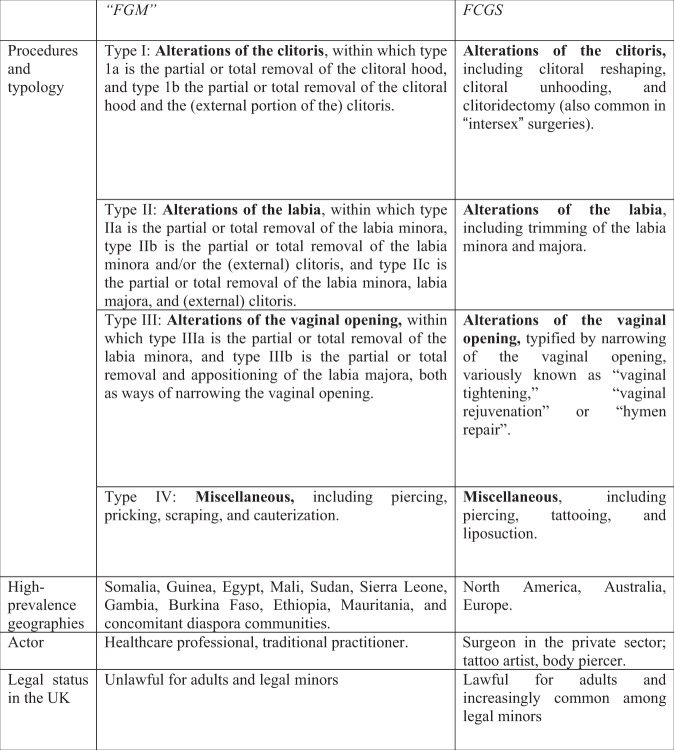
Comparing “FGM” and FCGS: examining the similarities and differences between the physical procedures, typology, geographies, pracitioners, and legal standing of the two practices. Adapted from [7], with references omitted.

Elsewhere, writing with Brian Earp, I have mapped the overlap between FGC and FGCS, comparing each component of the World Health Organization typology [6] of “female genital mutilation” to an equivalent practice within the category of FGCS [7]. Fig. 1 is an adapted version of a table from that work.

As Fig. 1 demonstrates, for every surgical procedure within the “FGM” typology, there is an equivalent modification practiced as FGCS. This collapses the received wisdom that FGC and FGCS are so different in their physical instantiations as to mandate differential treatment in medicine, public discourse, and the law. Indeed, the extent of their similarities calls into question the sense of maintaining two separate categories, a point to which I return later in the paper.

It might be objected that there are other, *non-physical* reasons to treat FGC and FGCS separately, and that we should instead focus on the intentions and meanings of the practices. (After all, stabbing a person in the street is not the same as making a surgical incision, though there may be some physical resemblance). I will now discuss some arguments that may be given in defence of the separate treatment of FGC and FGCS, and will show why these attempts to ground a significant difference between the practices do not succeed.

### Objection 1: “FGC is patriarchal”

In some contexts, FGC is practised with the explicit intention of curtailing female sexual pleasure or reducing promiscuity. However, this is only one among many motivations that vary considerably across the many disparate contexts, cultures, and practices of FGC. Amongst the many reasons for practicing FGC are the belief that cut genitals confer “normal” adult sexual identity, are more hygienic, represent ritual purification, are aesthetically pleasing, and that the practice satisfies a religious requirement [8].

Further, there is no community globally that practices female genital cutting and does not also practice male genital cutting—though many communities only practice male genital cutting—and the motivations for the two are often similar or identical. Indeed, the disciplining of sexuality is also sometimes a motivation for cutting male genitals [7]. Yet in most Western jurisdictions, male genital cutting is lawful at any age and with little or no regulation, while female genital cutting is unlawful at any age.

If the desire to challenge patriarchal practices is the reason for the draconian approach to FGC, FGCS should not get off so lightly. FGCS is widely acknowledged to be motivated by adherence to the aesthetic ideals portrayed in the highly-visible, increasingly hairless vulvae displayed in pornography [9], in which labia are minimal, and/or the pursuit of a “tight receptacle for penile penetration,” designed to accentuate penile pleasure [10]. FGCS, like many other body modifications marketed to Western women, plays on the idea that the unmodified female body is ugly, unfeminine, and faulty. As Camille Nurka puts it: “cosmetic labiaplasty is merely another proposed solution, in the long history of gynaecological medicine, to female sexual deficit thought to spring from a woman’s own defective body” [11].

### Objection 2: “FGC is unsafe”

In the UK, FGCS is exclusively performed in clinical settings, and is therefore liable to be safer than practices that are excluded from clinical settings by criminalisation. Even so, FGCS is inadequately regulated in most contexts, including in the UK [12], and there is a paucity of empirical data on the outcomes and complications—both physical and psychological—of FGCS procedures [13]. As such, it is difficult to compare the risks of FGC and FGCS procedures under equivalent conditions.

FGC is often represented as a procedure performed in rural, non-clinical settings with unsanitary instruments. Such depictions dovetail with Western conceptions of FGC as a “barbaric” practice carried out by “backward” peoples. In fact, a large and growing proportion of FGC in high-prevalence settings is performed by surgeons under clinical conditions, in much the same manner as FGCS [14]. This ought to be unsurprising: prevalence rates are upward of ninety percent in some contexts, and no parent would tolerate a perilous procedure for their child where safer options are accessible.

Even when performed in clinical settings, the more serious practices within both FGC and FGCS are high-risk surgeries, with many possible (and overlapping) complications. While traditional cutters working in non-clinical settings are usually highly-skilled, it is obviously more difficult to manage complications outside clinical settings. However, this may be taken as an argument in favour of medicalisation rather than outright prohibition, which tends to drive practices underground, leading to greater risk [15].

Third, no nonmedical bodily modification is completely risk-free, yet many are readily tolerated: i.e. body piercing, tattooing, orthodontics, laser hair removal, indoor tanning. It may be that there is an acceptable threshold for risk, but that would need to be determined by consulting empirical data on outcomes and complications and making careful arguments based on those facts. But that data is not readily available, careful arguments have not been made, and regardless, a risk threshold could not feasibly rule out all FGC procedures while supporting all physically-comparable FGCS procedures. Further, a consistently-applied risk-threshold would likely also outlaw nonmedical male genital cutting and intersex surgery.

In the absence of an evidence-based threshold and consistent regulation, distinctions based on “safety” are unfounded, and are liable to act as cover for more dubious bases for delineating the two practices.

### Objection 3: “FGC affects children”

FGC is often, though not always, performed on children. In some cultures, FGC is carried out in adolescence, and is precisely the act that confers adulthood, which poses a challenge to Western conceptions of childhood and adulthood and their relationship to the acquisition of the capacity to consent [16]. It is also increasingly common for FGCS to be performed on adolescents, and thousands of minors have undergone labiaplasty over the last decade in Western contexts [17]. Intersex surgeries and male genital cutting procedures are almost exclusively performed on infants, with little public or legal concern or condemnation compared with FGC [18].

If the reason for outlawing FGC is to protect children, who are unable to meaningfully consent to an irreversible bodily modification, then the prohibition should presumably shield them from *all* nonmedical surgeries. And if current FGC legislation is designed to protect children, then its current extension to adults is not justifiable, or, at least, requires separate justification. Current legislation in England, Wales, and Northern Ireland refers throughout to “a girl” and then later notes that “Girl includes woman,” thereby explicitly announcing its intention to depart from the usual legal norms and treat (particular) women as lacking the consent capacities that are usually afforded to adults [4].

The demand for consistency regarding the inability of children to consent to nonmedical bodily alterations would of course have serious consequences. All forms of nonmedical male genital cutting and intersex surgery would be unacceptable until the subject acquired the capacity to give consent. This would outrage many parents, and would require engagement with longstanding ethical questions as to when a person acquires the ability to give consent, how this ability is determined, and to what extent strong cultural norms might preclude meaningful consent for adults as well as young people.

### Objection 4: “FGC is not a choice”

One might argue that FGC is cultural, and culturally-mandated practices often do not feel “optional,” especially if membership within a community depends upon them, so that the possibility of consent is precluded. FGCS is, by contrast, often seen as “merely” cosmetic, and cosmetic practices relate to personal aesthetics, and are therefore more voluntary. As Virginia Braun puts it, FGCS is often framed as a “a practice *for the self*—an act you can choose as part of a general project of improvement of the body/self” (7, original emphasis). Yet this dichotomy cannot be maintained. Granted, where FGC is practised, it is usually practiced by the majority of people within a particular community in accordance with a powerful cultural norm, while FGCS is practised by only a small number of people within communities in which there is no cultural expectation of genital modification. (Though note that those who choose FGCS may belong to subcultures—e.g. online communities—in which the prevalence and pressure surrounding FGCS is significant [9]).

It must also be acknowledged that there is a forceful set of norms in operation more broadly which encourage women to “feminise” their bodies through various forms of bodily modification (by dieting, removing body hair, undergoing breast implantation etc.). FGCS clearly lies on this continuum [19]. Women’s “choices” in most societies globally are, as psychologists Moran and Lee put it “increasingly limited by narrow, heterosexist and homogenised representations of female sexual being” [20]. It is a stretch to claim that the practices on that continuum are entirely voluntary, given that social and sexual acceptance can depend upon how closely a woman approximates certain ideals, and violations are often met with social punishment. (Consider that it is still astonishingly rare to see a barelegged woman with visible leg hair.) In short, as Crouch et al. note, it’s “difficult to see how FGCS could be anything other than cultural” [21].

Not only is FGCS a “cultural” practice, FGC is often a “cosmetic” one; the dichotomy is much less clear than its adherents pretend. In contexts in which FGC has traditionally been practiced, the reasons for its continuation are increasingly focussed on cosmetic factors. In Egypt, the rates of FGC have risen in recent years, and health-workers performing the procedures increasingly understand their work as a cosmetic enhancement. In a recent study, one doctor said “I don’t call it circumcision, I call it ‘refinement’ […] you are beautifying the labia. It’s normal” [14].

Further, if the prohibition on FGC is justifiable because a near-inviolable cultural norm dictates its practice, which more or less rules out the possibility of it being chosen, even by adult women, then male genital cutting—a practice which modifies the genitals of infants who cannot consent, in order to meet a near-inviolable cultural norm—should also be prohibited on those grounds. Clearly this alone cannot explain the differential discourse regarding FGC and FGCS.

The cultural-cosmetic and voluntary-coerced distinctions are drawn far too quickly, and cannot straightforwardly ground the distinction between FGC and FGCS. The question of consent to such practices, even for adults, is a vexed one that philosophers contend with in their engagement with the problems of “false consciousness,” “adaptive preferences,” and “damaged autonomy” [22, 23].

## Why the inconsistency?

My arguments so far can be summarised as follows: FGC and FGCS not only have significant overlap in terms of their physical details, both practices are also risky, often performed on minors, and are motivated by cultural pressures, some of which are unambiguously patriarchal. Treating FGC and FGCS differently despite these social and physical similarities indicates that the real driver is how the *people* who practice them are perceived.

Western women are seen as rational agents, making their own choices about their bodies in a society in which patriarchal norms are weak or absent. Women of colour are instead seen as victims of their cultures and their menfolk, unable to make choices about their bodies due to the combined force of patriarchal pressures and the limiting cultures in whose shadow they live. One of the ways in which the “West” distinguishes itself from the rest of the world is via the myth that Western societies are either culture-free, or are governed by “civilised” cultures premised on autonomy and rationality, within which each person is able to make her own decisions. By contrast, “non-Western” cultures have long been portrayed as “backward,” “uncivilised,” “barbaric” places, saturated with cultures which prevent their inhabitants—and especially their women—from acting autonomously or rationally [19].

The dichotomy between the consenting white woman and the subjugated woman of colour is not only false, it is racist. Yet existing attempts to differentiate FGC from FGCS invariably rely upon, and reproduce, this distinction between white, Western women and girls, whose decisions are respected as instances of the freedom to make choices about their bodies, and non-Western women and girls, most of whom are people of colour, who are deemed to require the intervention of benevolent Western actors in order to be saved from cultures which harm them [24]. The plot thickens when one notes that the toleration of a person’s choices about their body, including choices that may seem unwise, is often presented as a uniquely Western virtue, and a justification for imposing “Western values” on others.

Not only do these representations themselves oppress women of colour and further limit their decisional space, but they prevent those in Western settings from critically analysing their own cultural practices. As Gayatri Chakravorty Spivak writes, it is important that we are able to ask “who is the other woman? How am I naming her? How does she name me?” [25]. Western commentators have concentrated their energies on criticising FGC, and making a spectacle of the practice. Perhaps it is time they turned the lens back on their own cultural practices, and started to consider the constructive dialogue that might be possible if only they could move beyond this divisive and indefensible binary. It is disappointing to note that similar pleas were made in the medical ethics literature fifteen years ago [26].

## Conclusion

I have argued that there is no distinction, physical, cultural, or otherwise, that can justify the differential treatment of FGC and FGCS in legal and public discourses. The current distinction appears to be made on the basis of the race, culture, or nationality of the girl or woman in question, which draws on, and entrenches, racism and ethnocentrism. The categories should be collapsed, and genital surgeries should be managed on a case-by-case basis, with attention to standard medical considerations, like age and level of risk. Professionals who regularly encounter FGC and FGCS would benefit from more consistent messaging, rather than being required to fall in line with a discriminatory, two-tier system [27].

I will finish by noting that while the distinction between FGCS and FGC is indefensible and should be scrapped, there is an obviously defensible distinction that should be consistently applied: that between those who have the capacity to consent to genital cutting, and those who do not [18]. I do not have space here to address the vexed question of whether consenting adults should be permitted to undergo bodily modifications of their choosing on request. But two things seem clear. First, making race, ethnicity, or nationality the determining factor is morally indefensible. Second, anyone *without* the capacity to consent to nonmedical genital modification—including intersex and male genital surgeries—should be protected from irreversible bodily changes until such a time as they are able to express their own wishes.
